# *OsLEA3-2*, an Abiotic Stress Induced Gene of Rice Plays a Key Role in Salt and Drought Tolerance

**DOI:** 10.1371/journal.pone.0045117

**Published:** 2012-09-14

**Authors:** Jianli Duan, Weiming Cai

**Affiliations:** 1 Institute of Plant Physiology and Ecology, Shanghai Institutes for Biological Sciences, Chinese Academy of Sciences, Shanghai, People’s Republic of China; 2 Graduate University of the Chinese Academy of Sciences, Beijing, People’s Republic of China; Kansas State University, United States of America

## Abstract

Late embryogenesis abundant (LEA) proteins are involved in tolerance to drought, cold and high salinity in many different organisms. In this report, a LEA protein producing full-length gene *OsLEA3-2* was identified in rice (*Oryza sativa*) using the Rapid Amplification of cDNA Ends (RACE) method. *OsLEA3-2* was found to be only expressed in the embryo and can be induced by abiotic stresses. The coding protein localizes to the nucleus and overexpression of *OsLEA3-2* in yeast improved growth performance compared with control under salt- and osmotic-stress conditions. *OsLEA3-2* was also inserted into pHB vector and overexpressed in *Arabidopsis* and rice. The transgenic *Arabidopsis* seedlings showed better growth on MS media supplemented with 150 mM mannitol or 100 mM NaCl as compared with wild type plants. The transgenic rice also showed significantly stronger growth performance than control under salinity or osmotic stress conditions and were able to recover after 20 days of drought stress. *In vitro* analysis showed that OsLEA3-2 was able to protect LDH from aggregation on freezing and inactivation on desiccation. These results indicated that *OsLEA3-2* plays an important role in tolerance to abiotic stresses.

## Introduction

Late embryogenesis abundant (*LEA*) genes code for a diverse group of proteins that accumulate to high levels in seed development [1]. LEA proteins play an important role in the maturation process of embryos and the accumulation of LEA proteins in embryos is correlated with the ability to tolerate desiccation [2]. The expression of *LEA* genes can be induced by the application of abscisic acid and by various abiotic stresses such as dehydration, osmotic stress, or cold in both reproductive and vegetative tissues [3], [4], [5], [6], [7], [8].

LEA proteins of 5–9 different homology classes have been categorized based on similarities in their amino acid sequences and expression patterns [5], [9], [10], [11]. The group of 3 LEA proteins are principally composed of tandem repeats of an 11-mer amino acid motif (TAQAAKEKAGE), forming an amphiphilic helical structure [12]. Based on their structure characteristics, the group 3 LEA proteins have been suggested to function in binding or the replacement of water, in the sequestration of ions, and in the preservation of protein and membrane structure during dehydration [5]. Group 3 LEA proteins, which are mostly cytosolic proteins [13], have been intensely analyzed in barley (*Hordeum vulgare*) [14], wheat (*Triticum* sp.) [15], cotton (*Gossypium* sp.) [12], rice (*Oryza sativa)* [7], maize (*Zea mays*) [16], and so on. The accumulation of the group 3 LEA transcript has been associated with genotypic differences in the acclimation to cold in rice varieties [17]. The expression of a group 3 LEA protein gene (*HVA1*) from barley has been shown to confer tolerance to water- and salt-stress in transgenic rice [18] and in transgenic mulberry [19]. Overexpression of a soybean group 3 LEA gene (*PM2*) confers the tolerance of in *E. coli* and stabilizes enzyme activity under diverse stresses [20].

Here, we report the isolation and functional characterization of the *OsLEA3-2* gene (accession no. JQ043381), which encodes a group 3 LEA proteins in rice. A semi quantitative RT-PCR analysis revealed that the *OsLEA3-2* gene does not express in vegetative tissues under normal conditions; however, it can be induced by abiotic stresses. To better understand the function of the OsLEA3-2 protein exposed to stresses conditions, *S. cerevisiae* cells that expressed *OsLEA3-2* were produced and the growth was compared with *S. cerevisiae* cells harboring a pYES2 vector under salt and osmotic conditions. The OsLEA3-2 cells displayed improved growth as compared to the control strain under salt- and osmotic-stress. *OsLEA3-2* overexpressing transgenic *Arabidopsis* and rice showed significantly stronger growth performance than control under abiotic stresses. OsLEA3-2 was also able to protect lactate dehydrogenase (LDH) from aggregation on freezing and inactivation on desiccation *in vitro*. All this results indicate that *OsLEA3-2* is conservative in resistance to abiotic stresses.

## Materials and Methods

### Plant Material and Treatments

The rice cultivar ZH11 was used in the transformation. Rice plants were grown in the glasshouse at 28°C and the *Arabidopsis* plants were grown in growth chambers at 21–25°C, 16 h/8 h (day/night).

To impose the salt and osmotic stresses, one month old seedlings of ZH11 were irrigated with 200 mM NaCl and 25% PEG-6000 or 400 mM mannitol in half strength Hoagland solution, respectively. For the ABA treatment, 100 µM ABA in 0.02% Tween-20 was sprayed on the ZH11 seedlings. One month old seedlings were incubated at 4°C for the cold treatment trials. All abiotic stress treatments, samples were harvested after 6 hours to analyze the expression of *OsLEA*.

### RNA Extraction and Reverse PCR

Total RNA was extracted from the embryo, root, shoot base, and leaf of the rice cultivar ZH11 using a TRIzol reagent (Invitrogen). Total RNA was also extracted from the control and stress-treated roots, shoot bases, and leaf tissues. The RNA was then reverse-transcribed using an oligo(dT)_18_ primer and AMV reverse transcriptase (Toyobo) according to the manufacture’s protocol. RT-PCR was conducted with oligos OSLEA2F and OSLEA2R (Table S1) using a Taq DNA polymerase (Dingguo) supplemented with 5% dimethyl sulfoxide (DMSO) to analyze the gene expression.

### Cloning and Sequencing of the Full-length *OsLEA* cDNA

The total RNA isolated from 200 mM NaCl treated seedlings was used to clone the *OsLEA* gene. The full-length *OsLEA* cDNA was cloned by 5′- and 3′-Rapid Amplification of cDNA ends (RACE), using a GeneRacer kit (Invitrogen) according to the manufacture’s protocol. The primer sets used in the 5′- and 3′-rapid amplification of cDNA ends experiments are listed in Table S1. PCR was performed using a PrimeSTAR DNA polymerase (TaKaRa) with a GC buffer, and the products of which were then cloned into a pMD18-T vector (TaKaRa) and sent to be sequenced.

### Subcellular Localization of OsLEA3-2

Onion epidermal cells were bombarded with the construct of a double *35 S: GFP: OsLEA3-2* transgene using a particle gun-mediated system (PDS-1000/He; Bio-Rad) and were observed with a confocal microscope (Zeiss LSM510; Carl Zeiss MicroImaging GmbH, Jena, Germany).

### Overexpression of OsLEA3-2 in Saccharomyces Cerevisiae

*OsLEA* was amplified with the following primers, OsLEA-F: CCC AAG CTT AAA ATG GCG TCG AGG CAG GAC A and OsLEA-R: TGC TCT AGA TCA CAT CTT GCC GTG GCG primers. *Hind* III and *Xba* I restriction sites are underlined in the forward and reverse primers, respectively. The amplicon was digested with *Hind* III and *Xba* I and placed under the control of a *GAL1* promoter in the plasmid pYES2. To achieve high levels of expression of *OsLEA* in *Saccharomyces cerevisiae*, the resulting plasmid, pYES2OsLEA, was transferred into the *S. cerevisiae* strain Y001582 using the lithium acetate method. Cells were cultured in a YNPD medium (0.67% yeast nitrogen base without amino acids, and 2% glucose) or in a YNBG medium (0.67% yeast nitrogen base without amino acids, and 2% galactose).

For the ionic and osmotic stress assay, *S. cerevisiae* cells that contained pYES2OsLEA were grown in YNBG medium for two days. Two hundred microliters of the culture was inoculated into YNPG (20 ml) supplemented with 1.2 M NaCl, 1.2 M KCl, or 2.0 M sorbitol. At each time point, 50 microliters of culture was removed, and the OD_600_ was measured with a spectrophotometer. Growth was measured three times.

### Overexpression of *OsLEA3-2* in *Arabidopsis*

*OsLEA3-2* was cut from pYES2-OsLEA3-2 with *Hind* III and *Xba* I and cloned into modified binary vector pHB [21]. The binary vector pHB-OsLEA3-2 was transformed into the *Agrobacterium* strain *EHA105* and was used for transformation into *Arabidopsis thaliana* ecotype Columbia according to a previously described method [22]. The transgenic plants were then selected on hygromycin. The T1 plants were confirmed by PCR with specific primers for the hygromycin phosphotransferase (*HPT*) gene: 5′-TGTCCTGCGGGTAAATAGC-3′ and 5′-TGCTCCATACAAGCCAACC-3′ (AY836546). To evaluate the abiotic stress tolerance of transgenic plants over expression of *OsLEA3-2* in the germination stage, wild type (WT) and transgenic (T4) seeds were sown on a MS, MS+150 mM mannitol or an MS+100 mM NaCl media.

### Ectopic Expression of *OsLEA3-2* in *Oryza Sativa*

The binary expression construct pHB-OsLEA3-2 was introduced into *japonica* rice Zhonghua 11 by an *Agrobacterium* mediated transformation as previously described [23]. Southern blot was used to analyze the transgenic plants. 20 µg of total genomic DNA from leaf tissue of transgenic plants and wild type plants was digested with appropriate restriction endonuclease *Hind* III. DNA fragments were separated by electrophoresis on a 1% (w/v) agarose gel and then transferred to a nylon membrane (Amersham Bioscience) according to standard protocols. Dig-high DNA labeling kit I (Roche) was used to label the *Hygromycin* DNA probe. Transgenic rice (*OsLEA3-2* overexpression) seeds were used for stress tolerance assays. Rice seeds were germinated in water (as control) or in water containing 100, 200 mM NaCl, 10%, 20% PEG 6000, or 10 µM ABA. Photographs were taken after five days and 14 days.

### Drought Stress Treatment

Both the wild type and transgenic rice plants were placed in soil and allowed to germinate and grow for one month in a glasshouse at 28°C±1°C. The one month old seedlings were then treated for drought stress (without irrigation) for 20 or 18 days. The plants were then irrigated with water and grown for one month and ten days. The plants that survived developed new leaves were counted.

### Aggregation Assay

LDH was obtained from SIGMA and diluted in 100 mM sodium phosphate buffer (PBS, pH6). Aggregation of LDH was monitored by reading absorbance *A* at 340 nm in a NanoVue Plus spectrophotometer. 170 µl of 0.2 mg/ml LDH with or without 0.4 mg/ml protectant proteins in polystyrene tubes was flash-frozen by immersion in liquid N_2_ for 30 s and then thawed at ambient temperature. All samples were assayed in triplicate.

### LDH Activity

LDH enzyme activity was measured according to Goyal, K. [24] with modifications. 2 µl of 0.1 mg/ml LDH was added to a total of 50 µl of 25 mM Tris/HCl (pH7) with or without stabilizing agents in a microfuge tube. Tubes were vacuum-dried for 1 hour at 25°C in an Eppendorf vacuum dryer and rehydrated immediately in 50 µl of water at room temperature. 2 µl of LDH/stabilizer solution was added to 1 ml of 100 mM PBS (pH6) with 100 µl NADH and 2 mM pyruvate. Change in A340 was monitored every 5 s for 1 min in a NanoVue Plus spectrophotometer. All values given are expressed as percentage of rate of reaction measured for undried samples. Assays were performed in triplicate.

## Results

### Cloning and Characterization of the Full-length cDNA of *OsLEA3-2*

**Figure 1:**
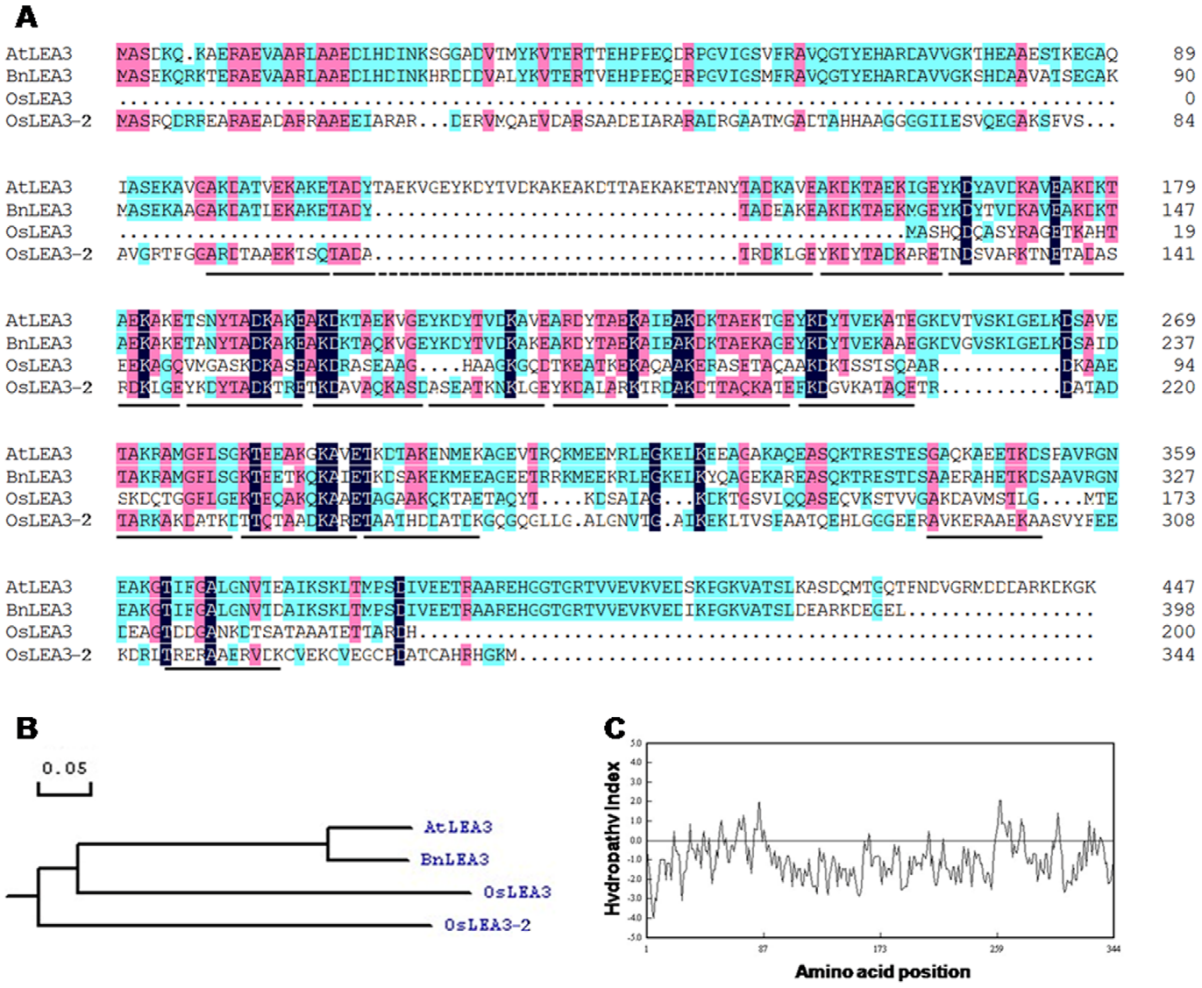
*In silico* analysis of the OsLEA3-2 protein. (A) Comparison of deduced amino acid sequence of OsLEA3-2 with OsLEA3 (AAD02421.1) and LEA3 proteins from *A. thaliana* (BAA11017.1) and *B. napus* (ACJ39155.1), underlines show the 11-mer repeats in the OsLEA3-2; (B) phylogenetic analysis of these group 3 LEA proteins; (C) hydropathy analysis of OsLEA3-2 protein.

The full-length cDNA was obtained by 5′ and 3′ Rapid Amplification of cDNA ends (RACE). The reverse specific primers oligo1 and oligo2, together with GeneRacer primers, Race 5′ and Race 5′ nested primer, were used to clone the 5′ end of the cDNA sequence. The forward specific primers oligo3 and oligo4 together with Race 3′ and Race 3′ nested primers were used to clone the 3′ end cDNA sequence (Table S1). The complete cDNA sequence was 1298 bp in size, with a 96 bp 5′ untranslated region (UTR) and a 167 bp 3′ untranslated region (Fig. S1). It consisted of two exons, encompassed one intron, and potentially encoded for an ORF of 344 aa with a predicted molecular mass of 36.8 kD and pI of 6.81. The deduced amino acid sequence shares 42 and 36% amino acid identities with LEA3 of *Arabidopsis thaliana* and *Oryza sativa* (Fig. 1A, B), respectively. This gene was termed *OsLEA3-2* and its accession number at GenBank was JQ043381. Predicted proteins of *Oryza* LEA3-2 showed a preponderance of Ala, Thr, Asp, Glu, Lys, Arg and Gly that constitute 20.6%, 10.2%, 10%, 9.3%, 9.3%, 9.3%, and 7.3%, respectively, and lack Trp. OsLEA3-2 has 16 11-mer repeating units, TKDA(A/T)ADK(A/T)RE (Fig. S2). Hydropathy analysis showed that OsLEA3-2 is a hydrophilic protein (Fig. 1C).

### *OsLEA3-2* Only Expresses in the Embryo Under Normal Conditions

**Figure 2:**
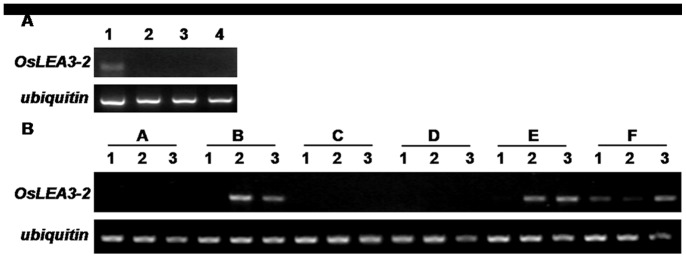
*OsLEA3-2* gene expression. (A) Expression profile of *OsLEA3-2* in different tissues of *Oryza sativa.* Lane 1, embryo; lane 2, root; lane 3, shoot base; lane 4, leaf. (B) RT-PCR analysis of *OsLEA3-2* gene expression in *Oryza sativa* under different stresses. Irrigation with half-strength Hoagland solution (A, control), 400 mM mannitol (B), sprayed with 100 µM ABA in 0.02% Tween-20 on seedlings (C), 4°C incubation (D), 200 mM NaCl (E), 25% PEG-6000 (F). 1, roots; 2, shoot base; 3, leaves.

The expression profile of *OsLEA3-2* gene in various tissues was analyzed employing the semi quantitative RT-PCR method. The *ubiquitin* gene was used as an internal control. A product of approximately 146 bp was amplified from the late embryo stage, while no transcript was detected in the roots, shoot base, and leaves of another plant (Fig. 2A). These results suggested a similarly to the previous reported information on LEA proteins, and that OsLEA3-2 may play an important role in the maturation process of the embryo.

### Expression Analysis of *OsLEA3-2* Under Abiotic Stresses

A semi quantitative RT-PCR analysis with the *ubiquitin* gene as an internal control revealed that the *OsLEA3-2* gene does not express in vegetative tissues under normal conditions, however, it can be induced by abiotic stresses. Treatment of the seedlings with mannitol, salt, or PEG induced *OsLEA3-2* gene expression. (Fig. 2B). While no transcript was detected in the seedlings treated with low temperature stress or ABA, *OsLEA3-2* expression was detected when the rice was grown in Hoagland with ABA (Fig S3). *OsLEA3-2* was induced by the ABA, but assimilation was not possible through a topographical application on rice leaves. Mannitol and salt stress also induced *OsLEA3-2* gene expression in the shoot base and leaves, while the PEG treatment induced gene expression in both roots and shoots.

### Subcellular Localization of OsLEA3-2

**Figure 3:**
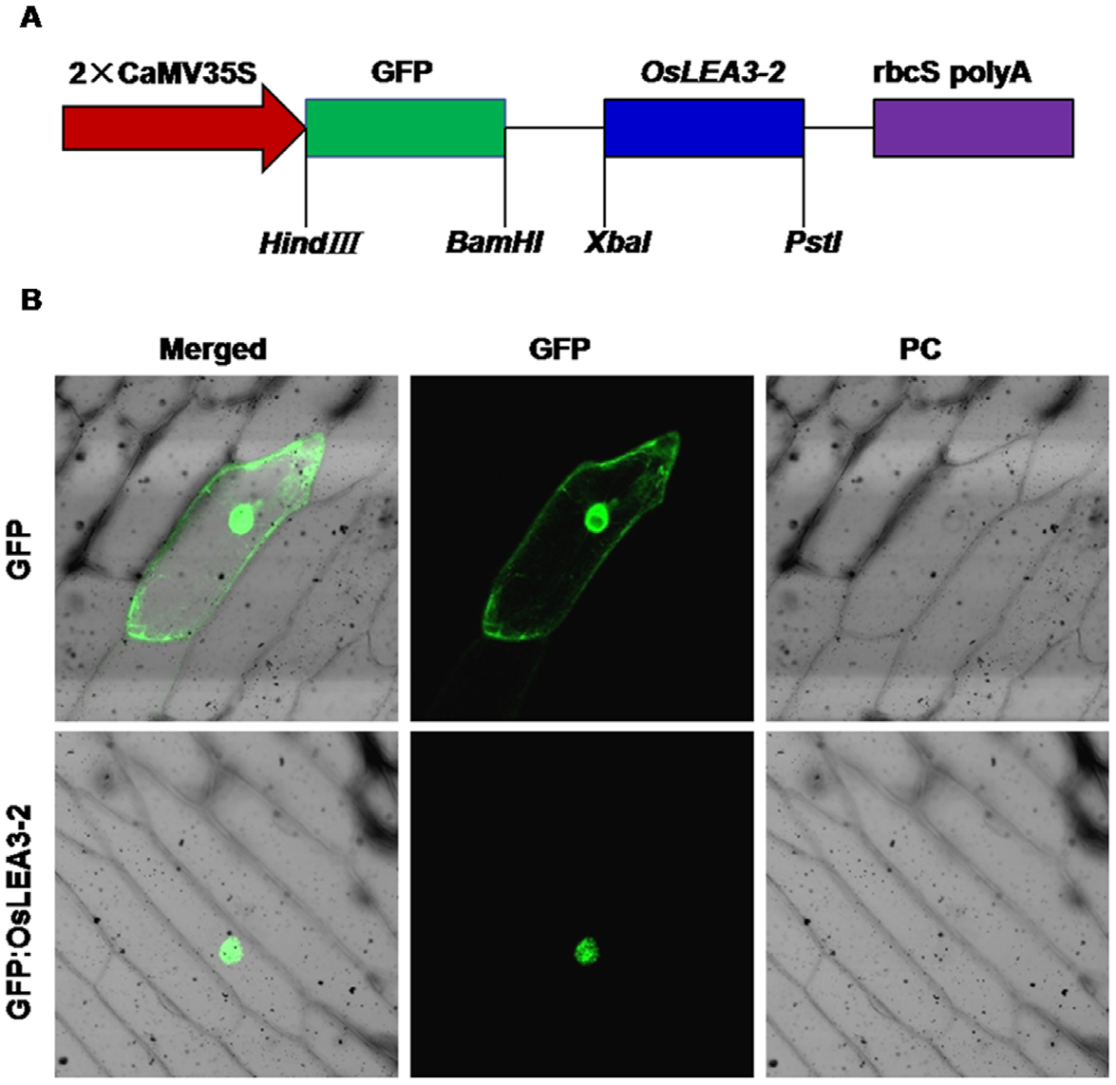
Subcellular localization of OsLEA3-2. (A) Diagram of the T-DNA region of the binary vector pHB::GFP::OsLEA3-2 used for transformation; (B) Subcellular localization of OsLEA3-2. The green fluorescent signal of GFP:OsLEA3-2 was detected exclusively within the nucleus of onion epidermal cells, while GFP by itself was detected both in the cytoplasm and nucleus. DIC (Differential Interference Contrast), referring to bright field images of the cells.

The *OsLEA3-2* coding sequence was fused in frame to the 3′ end of a GFP (Fig. 3A). The subcellular localization of the GFP-OsLEA3-2 was examined through a transient expression of *GFP-OsLEA3-2* in onion epidermal cells. The construct with *GFP* alone was used as a control. An examination of green florescence by confocal laser-scanning microscopy showed that GFP alone localized at the nucleus and cytosol of onion epidermal cells, while the green fluorescent signal of GFP:OsLEA3-2 was detected exclusively in the nucleus of the onion epidermal cells (Fig. 3B). More than 30 GFP positive cells were detected. GFP:OsLEA3-2 exhibited nucleus localization in all those cells. Another construct with *OsLEA3-2* coding sequence fused in frame to the 5′ end of a GFP was also used to determine the OsLEA3-2 subcellular localization. Nucleus concentrated green fluorescent signal was detected (data not shown). These results demonstrated that OsLEA3-2 is a nucleus- localized protein.

### Growth Performance of Transformed *S. cerevisiae* Strains Under Abiotic Stresses

**Figure 4:**
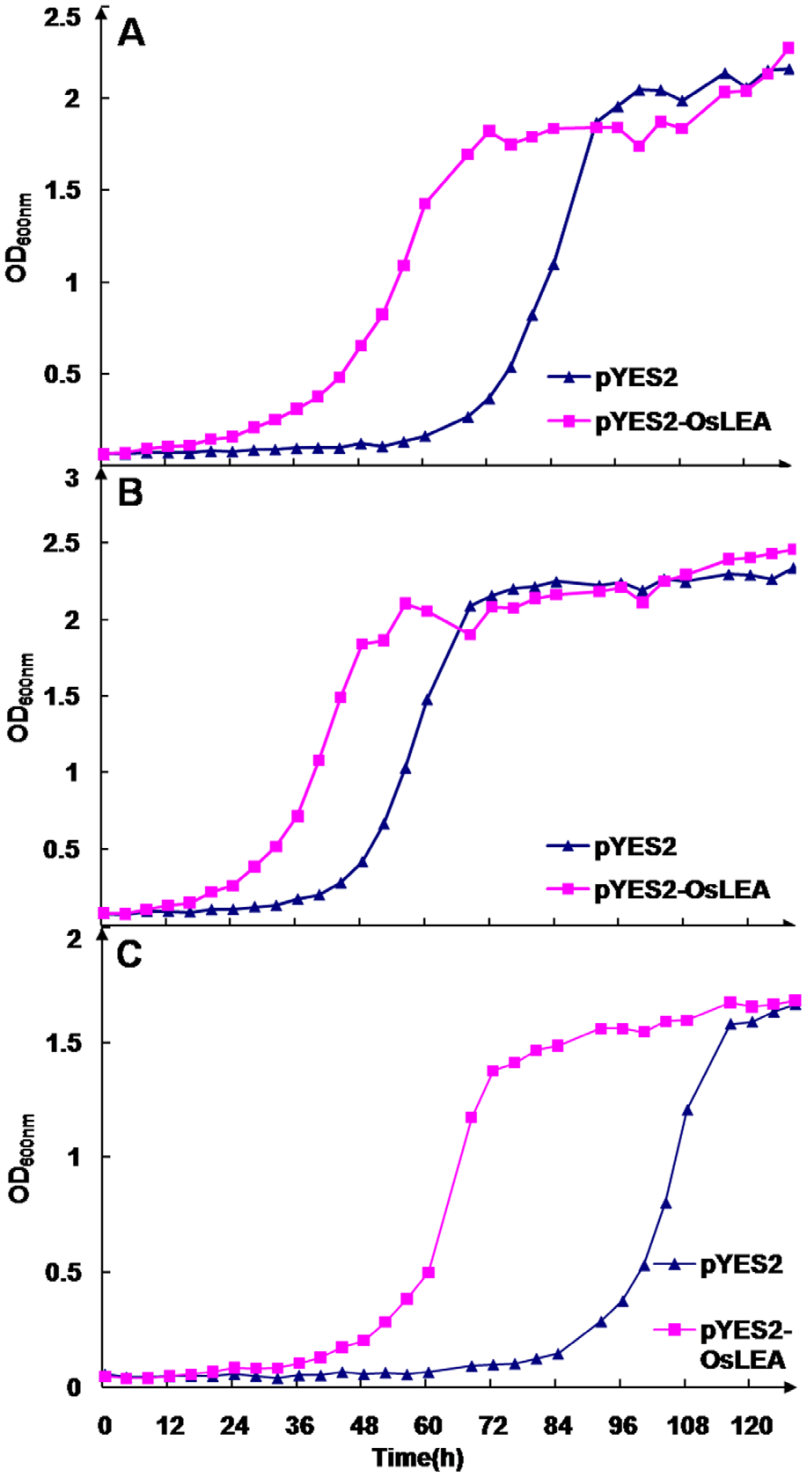
Growth of *S. cerevisiae* cells expressing *OsLEA3-2* and the control cell in the medium containing 1.2 M NaCl (A), 1.2 M KCl (B), and 2.0 M sorbitol (C). Yeast cells were grown on YNBG medium for two days. Twenty microliters of the culture was inoculated into YNBG supplemented with either 1.2 M NaCl, 1.2 M KCl, or 2.0 M sorbitol. At each time point, 60 microliters of culture was removed, and OD_600_ was measured with a spectrophotometer.

The growth performance of yeast cells transformed with pYES2 or pYES2-OsLEA3-2 was tested under ionic and osmotic stress conditions. The control strain, Y001582/pYES2, was grown in a YNBG medium to an early stationary phase and then transferred to a YNBG medium supplemented with 1.2 M NaCl. The growth of the Y001582/pYES2 strain was initially arrested and exponential growth resumed after a lag phase of 60 hours (Fig. 4A). The Y001582/pYES2OsLEA3-2 strain cells displayed improved growth as compared to the control strain, and the lag phase of Y001582/pYES2OsLEA3-2 was about 24 hours, shorter than that observed with the control when transferred to 1.2 M NaCl (Fig. 4A). Similar experiments were carried out to investigate the effect of high KCl concentration (1.2 M) or osmotic stress (2.0 M sorbitol) on growth characteristics. The Y001582/pYES2OsLEA3-2 cells displayed a shorter lag phase (24 h) than the control strain (39 h) in YNBG medium containing 1.2 M KCl (Fig. 4B). A similar phenomenon was observed in a YNBG medium supplemented with 2.0 M sorbitol. The Y001582/pYES2OsLEA3-2 strain again displayed a shorter lag phase (48 h) than the control strain (84 h) (Fig. 4C).

### *OsLEA3-2* Plays a Role in Abiotic Stress Tolerance of Plants

**Figure 5:**
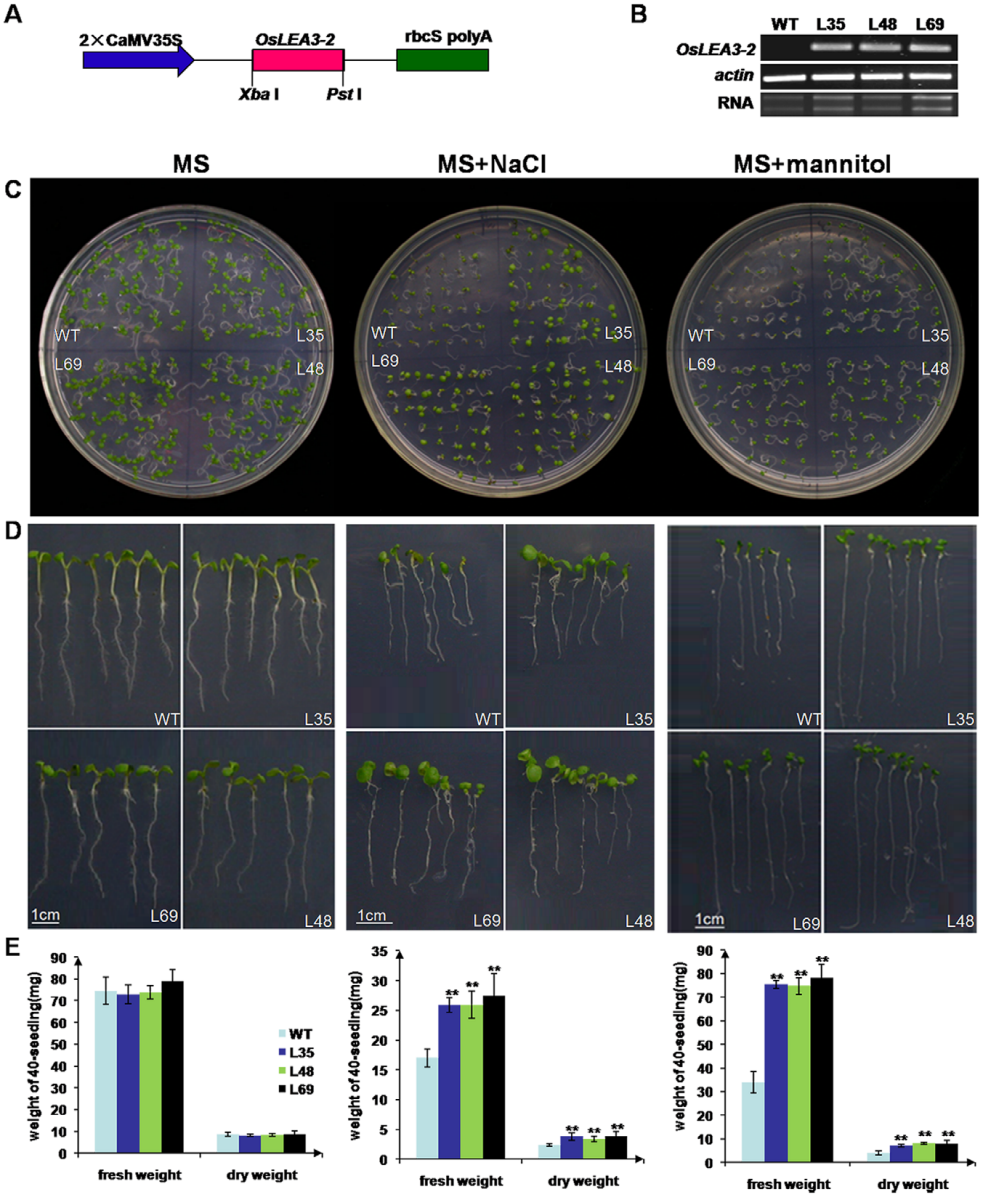
Effect of osmotic or salinity stress on *Arabidopsis* seedlings from wild type and *OsLEA3-2*-overexpressing transgenic lines (L35, L48, and L69). (A) Diagram of the T-DNA region of the binary vector pHB::OsLEA3-2; (B) *OsLEA3-2* expression level in transgenic *Arabidopsis*, wild type plants as control; (C, D) Sorbitol or NaCl sensitivity of wild type or transgenic seedlings. Photographs were taken following 9 days of growth on media containing 0 (control), 200 mM sorbitol, or 100 mM NaCl. (E) Fresh/dry weights of 9-day-old seedlings. All samples were measured in triplicate. Statistical significance was determined by Student’s *t* test. **P<0.01 shown above the bar reprent results significantly different from wild type control.

**Figure 6:**
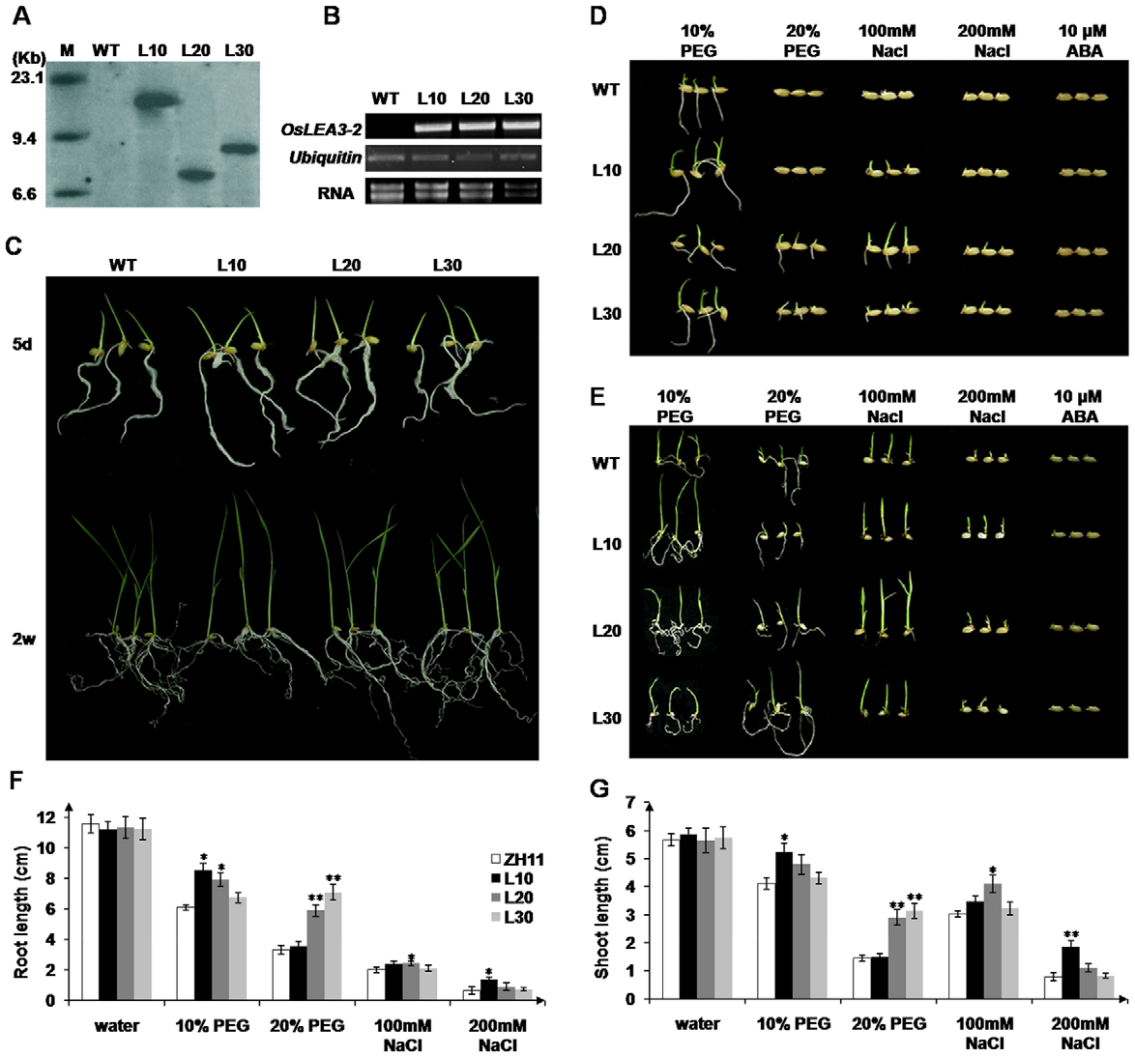
Stress or ABA sensitivity of wild type and transgenic rice seedlings. (A) Southern hybridization analysis of transgenic plants. M, molecular marker; WT, wild type; L10, L20, L30, three transgenic lines. (B) Expression level of *OsLEA3-2* in transgenic rice, Zhonghua 11 was used as control; (C) photographs were taken after 5 d or 2 weeks of growth in water control. Photographs were taken after 5 d (D) or 2 w (E) of growth in water containing 10% or 20% PEG, 100 or 200 mM NaCl, or 10 µM ABA. Effect of 2 weeks abiotic-stress on root (F) and shoot (G) length of rice. For each treatment, 8 seedlings were measured. *P<0.05 and **P<0.01 shown above the bar reprent results significantly different from wild type control.

To analyze the roles of *OsLEA3-2* in plants, *OsLEA3-2* was inserted into a pHB vector (Fig. 5A) and was overexpressed in *Arabidopsis* and rice under the control of the double constitutive cauliflower mosaic virus (CaMV) 35 S promoter. Transformants were selected according to hygromycin-resistance. Hygromycin gene specific primers were then used to perform PCR on genomic DNA from the transgenic lines or the wild type. Transgenic lines showed a 576 bp specific amplicon, while no observed size specific amplicon was amplified from WT plants. Stable inherited homozygous transgenic lines were obtained at the T2 generation. Three rice transgenic lines (L10, L20, and L30) were selected for Southern blot analysis. A single specific band was observed in each transgenic line (Fig. 6A). No band was observed in the wild type control. These results suggested single integration was occurred in the genome of each transgenic line. RT-PCR assays revealed *OsLEA3-2* was overexpressed in both the transgenic *Arabidopsis* and rice plants (Fig. 5B, 6B).

To assess the role of OsLEA3-2 in tolerance to abiotic stresses, transgenic *Arabidopsis* plants that overexpressed *OsLEA3-2* were compared with WT under abiotic stress conditions. No differences in seed germination percent were observed between the transgenic and WT plants under abiotic stress conditions, but a faster speed of germination was observed in the transgenic rice (Fig. S4). The *OsLEA3-2* overexpressing transgenic seedlings showed significantly stronger growth performance than WT plants (P<0.01) on MS media supplemented with 150 mM mannitol or 100 mM NaCl (Fig. 5C, D, E). The measurement of fresh/dry weight of seedlings revealed that the sorbitol stress had no clear effect on the transgenic lines, whereas it impaired the wild type growth (Fig. 5E).

Homozygous *OsLEA3-2* transgenic rice or wild type Zhonghua 11 seeds were soaked in water, 100 mM NaCl, 200 mM NaCl, 10% PEG 6000, 20% PEG 6000, or 10 µM ABA at 28°C. There was no difference in the growth performance between the *OsLEA3-2* overexpressing transgenic rice and the wild type Zhonghua 11 treated with water (Fig. 6C). The transgenic seedlings that overexpressed *OsLEA3-2* showed a stronger growth performance than the control under salinity or osmotic stress after 5 d (Fig. 6D). Even after 14 days, transgenic lines still showed higher root and shoot growth than those of the control plants (Fig. 6E). In 10% PEG, transgenic lines (L10 and L20) that overexpressed OsLEA3-2 showed a significantly stronger root growth performance than the control (P<0.05) (Fig. 6). Transgenic line L30 also had an improved growth performance. However, in 20% PEG, no striking difference was detected between transgenic line L10 and control ZH11, while L20 and L30 showed a significantly stronger growth performance than control (P<0.01). One of the three transgenic lines showed a significantly stronger growth performance than control under salinity stress, L20 in 100 mM NaCl (P<0.05) and L10 in 200 mM NaCl (P<0.05). Other lines showed a better growth performance (Fig. 6). No elevated growth performance of transgenic rice was observed under the ABA treatment and the transgenic line L20 showed the best growth performance under abiotic stress conditions.

### OsLEA3-2 is Able to Enhance Drought Tolerance in the Transgenic Rice Plants

**Figure 7:**
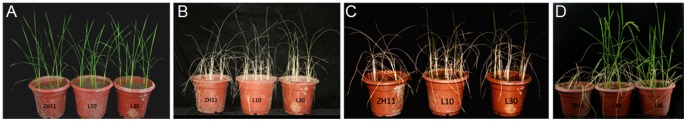
Drought-treatment assay of wild type and transgenic rice plants. One month old seedlings of the wild type cultivar and transgenic plants (A) were treated with drought stress (without irrigation) for 20 days (B), then irrigated with water and grown for 10 days (C), and one month (D). 1, wild type; 2, transgenic line 10; 3, transgenic line 30. Each container had six plants.

To examine whether the overexpression of the *OsLEA3-2* gene conferred resistance to drought stress, one month old transgenic rice seedlings from lines 10 and 30 were treated in drought stress conditions for 20 days (Fig. 7A). It was found that leaves of both the wild type and transgenic plants could not expand in size after 20 days of drought stress (Fig. 7B), indicating that the drought stress completely inhibited their growth. After 20 days without irrigation, the leaves appeared to be bleached and were senescent in both the wild type and the transgenic rice (Fig. 7B). However, new leaves appeared only in the transgenic plants after the plants were supplied with water and grown in optimal conditions for 10 days post stress (Fig. 7C). None of the wild type plants survived the stress conditions, while 83% of the plants from line 10 and 67% from line 30 survived. The transgenic rice plants grew until the flowering stage (Fig. 7D) and later set seeds (data not shown).

**Figure 8:**
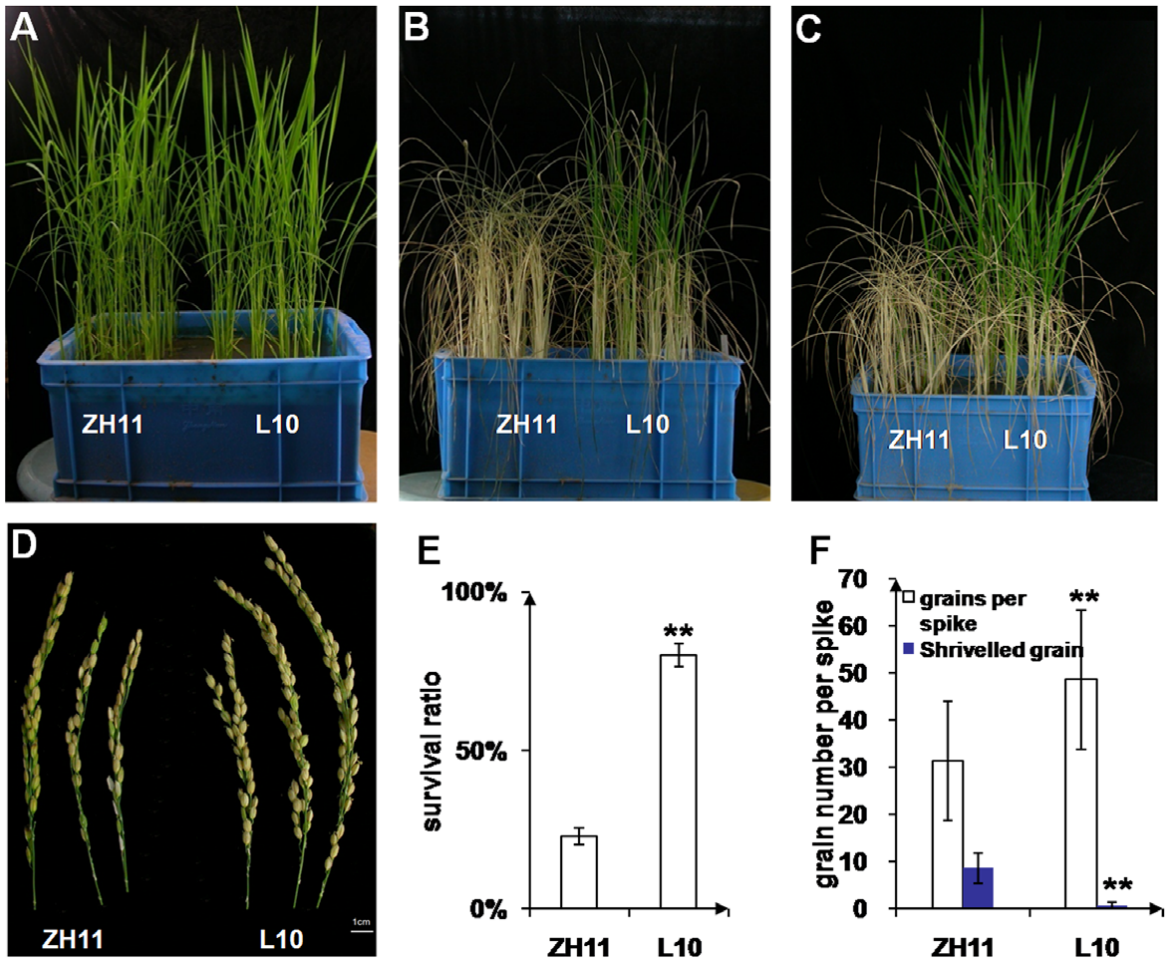
Effect of drought-stress on wild type and transgenic rice plants. One month old seedlings of the wild type cultivar and transgenic plants (A) were treated with drought stress (without irrigation) for 18 days (B), then irrigated with water and grown for 10 days (C). (D) Spikes from survived ZH11 and transgenic line L10. (E) Survival ratio of the rice plants. (F) Statistic analysis of the grains. Three and ten spikes were analyzed for ZH11 and transgenic line L10, respectively. **P<0.01 shown above the bar reprent results significantly different from wild type. Effect of drought-stress was assayed in triplicate.

About 20% wild type plants could survive on 18-day-drought stress. However, those plants produced less grains (with one third shriveled) per spike than transgenic line L10 (Fig. 8).

### OsLEA3-2 Protein Prevents LDH Aggregation Due to Freezing Stress

**Figure 9:**
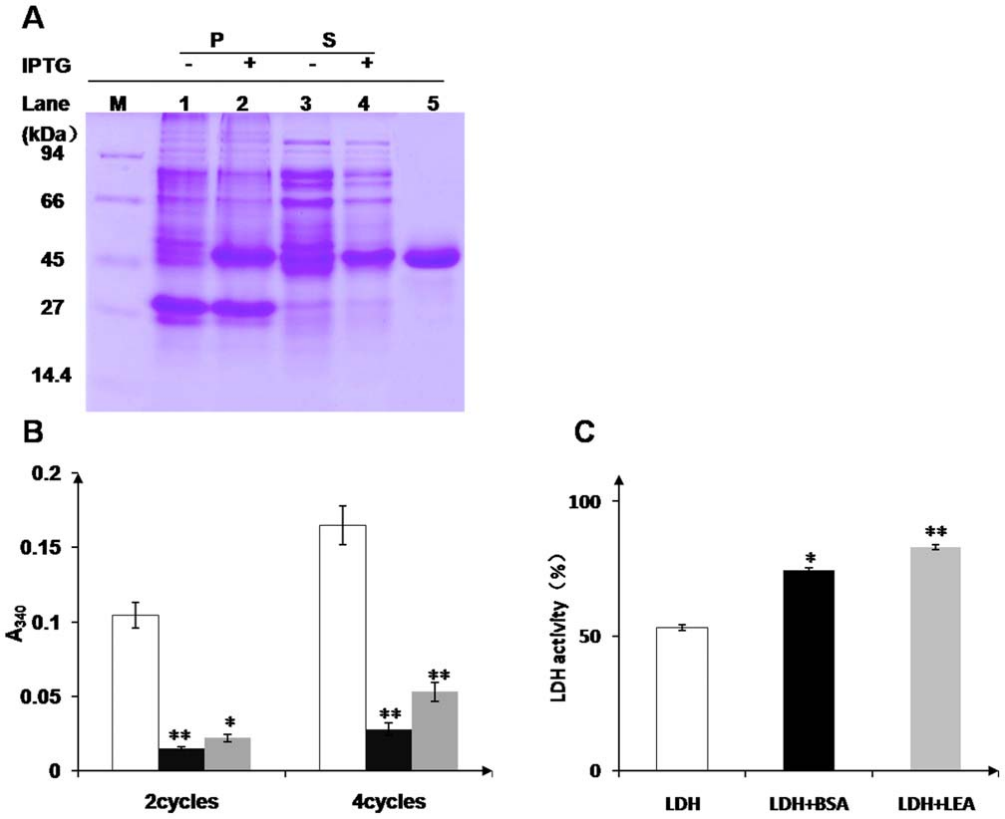
Protection of LDH by protein protectants. (A) SDS-PAGE analysis of OsLEA3-2 protein expression and purification. M, protein molecular weight marker; 1, 3, uninduced precipitant and soluble supernatant of BL21 (DE3) cells, respectively; 2, 4, induced precipitant and soluble supernatant, respectively; 5, OsLEA3-2 protein after purification. (B) Protection of LDH by protein protectants from aggregation due to freezing and thawing. LDH aggregation on repeated freezing and thawing is indicated by light scattering at A_340_. Results are shown for LDH alone (open bar), for LDH in the presence of BSA (black bar) and for LDH together with OsLEA3-2 (grey bar). *P<0.05 and **P<0.01 shown above the bar reprent results significantly different from those for LDH alone. (C) Effect of desiccation on LDH activity. LDH activity after vacuum drying (open bar) in the presence of BSA (black bar) or OsLEA3-2 (grey bar). All samples were assayed in triplicate.

LDH suffers marked aggregate formation when subjected to cycles of freezing in liquid N_2_ followed by thawing, as determined in a light-scattering assay. The degree of aggregation increases with the number of freeze-thaw cycles, but addition of OsLEA3-2 or BSA, a well-known cryoprotectant protein, prevents the aggregation (Fig. 9B). OsLEA3-2 protein prevents LDH aggregate formation due to rapid freezing in liquid N_2_.

### OsLEA3-2 Protein Preserves LDH Activity Under Desiccation

In accordance with its anti-aggregation activity, OsLEA3-2 preserves LDH activity under desiccation. BSA preserves enzyme activity to a lesser extent than OsLEA3-2 after multiple cycles of desiccation (Fig. 9C).

## Discussion

Plants have developed mechanisms to perceive and transmit stress signals to cellular machinery that active adaptive responses in response to abiotic stress conditions [25], [26]. It has been shown that LEA proteins are involved in plants drought/desiccation tolerance. LEA proteins are members of a large group of hydrophilic, glycine-rich proteins that are thought to function through the maintenance of protein or membrane structure, sequestration of ions, or as molecular chaperones to help prevent the formation of damaging protein aggregates [24], [27], [28]. In this study, we cloned a LEA protein gene *OsLEA3-2* in *Oryza sativa* and found it to be expressed in late embryo stage and could be induced by abiotic stresses. The OsLEA3-2 gene is located in the nucleus (Fig. 3), along with *MsLEA3-1* [29], while the cotton group 3 LEA protein D-7 is present only in the cytosol [13]. Different group 3 LEA proteins have different subcellular locations, which indicate that the group 3 LEA proteins have different functions.

The *OsLEA3-2* gene enhanced salt and osmotic stress tolerance when expressed in *Saccharomyces crevisiae*. Ectopic expressions of the LEA protein genes in *S. crevisiae* or *E. coli* improve the growth performance of the transformant under abiotic stresses [20], [30], [31], [32]. The growth performance of *S. cerevisiae* cells that expressed *le4*, *le25*, or *HVA1* [30], [31] was improved under salt stress conditions as compared with the control, however, no improved growth performance was observed under osmotic stress (sorbitol). *S. cerevisiae* cells expressing *OsLEA3-2* displayed an improved growth under both ionic and osmotic stress conditions (Fig. 4).

To elucidate the contribution of OsLEA3-2 to abiotic stresses, *Arabidopsis* transgenic plants with the *OsLEA3-2* gene overexpressed were generated. The transgenic lines were shown to be more tolerant than the wild type plants to salt and osmotic stresses (Fig. 5C). This suggests that LEA proteins from *Oryza sativa* (a monocot), can function properly in *Arabidopsis*, a dicotyledonous plant. There was no significant growth difference between the *Oryza sativa* transgenic plants overexpressing *OsLEA3-2* gene and wild type plants under field conditions with normal irrigation (Fig. S5). Three transgenic lines (L10, L20, and L30) exhibited the same phenotypes as wild type for the majority of morphological traits observed (e.g., plant height, leaf length, spike length, and 1000-seed weight). However, transgenic seedlings that overexpressed *OsLEA3-2* showed a significantly stronger growth performance than control under salinity or osmotic stress (Fig. 6D, E). The wild type rice could not survive the drought stress conditions, while transgenic plants that overexpressed *OsLEA3-2* recovered and grew until the flowering stage and set seeds (Fig. 7), indicating that the transgenic lines could tolerate 20 days of drought stress and the drought stress did not completely damage the fertility of the transgenic rice plants. These results demonstrate that transgenic rice plants that overexpress the *OsLEA3-2* gene are suitable for practical applications and are capable of the production crops even if exposed to long periods of drought stress.

LDH undergoes aggregation on freezing and inactivation on drying (Fig. 9). Both OsLEA3-2 and BSA are able to protect LDH from aggregation and inactivation due to stresses. BSA is a well-known cryoprotectant protein. Even though OsLEA3-2 protects LDH from aggregation on freezing and thawing to a lesser extent than BSA (Fig. 9B), it is more effective than BSA in protecting enzyme activity on desiccation (Fig. 9C). Other studies of LEA function have found various LEA proteins to have protective properties similar to or better than BSA on freezing [33], [34]. LEA proteins are more effective than BSA in protecting enzyme activity [24], [35] on desiccation. All those suggest that LEA proteins are able to maintain enzyme activity on dehydration.

## Supporting Information

Figure S1 The *OsLEA3-2* cDNA sequence and its deduced amino acid residues. The asterisk shows the stop code, the polyadenylation signal sequence (AATAAA) is underlined. The full length cDNA of *OsLEA3-2* is 1,298 bp in size, with a 96 bp 5′ untranslated region (UTR) and a 167 bp 3′ untranslated region, and encodes an ORF of 344 aa.
(TIF)

Figure S2 Summation of amino acids in the 11-mer repeating units. The upper number indicates the position of the amino acid, and the number in the parenthesis shows the frequency of the amino acid. A general consensus sequence, TKDA(A/T)ADK(A/T)RE, was determined.
(TIF)

Figure S3 The expression profile of *OsLEA3-2* in *Oryza sativa.* Lane 1, hongland; lane 2, hongland+10 µM ABA.
(TIF)

Figure S4 Plant height (A), spike length (B), leaf length (C), and 1,000-seed weight (D) of transgenic lines overexpressing *OsLEA3-2* and wild type zhonghua 11 under field conditions with normal irrigation. Twelve samples were measured for plant height, spike length, and leaf length of each line. 1,000-seed weight was measured in triplicate.
(TIF)

Figure S5 The germination of rice seeds in water (A), water+100 Mm NaCl (B), water+10 µM ABA (C), water+10% PEG (D) for 6 days. The germinated seed number was counted every day. The germination of rice seeds was assayed once.
(TIF)

Table S1 Primers.
(TIF)
