# Supplementary figures and images for: OsLEA3-2, an Abiotic Stress Induced Gene of Rice Plays a Key Role in Salt and Drought Tolerance
Source: PLoS One. 2012 Sep 14;7(9):e45117. doi: 10.1371/journal.pone.0045117 (PMC3443202; doi:10.1371/journal.pone.0045117)

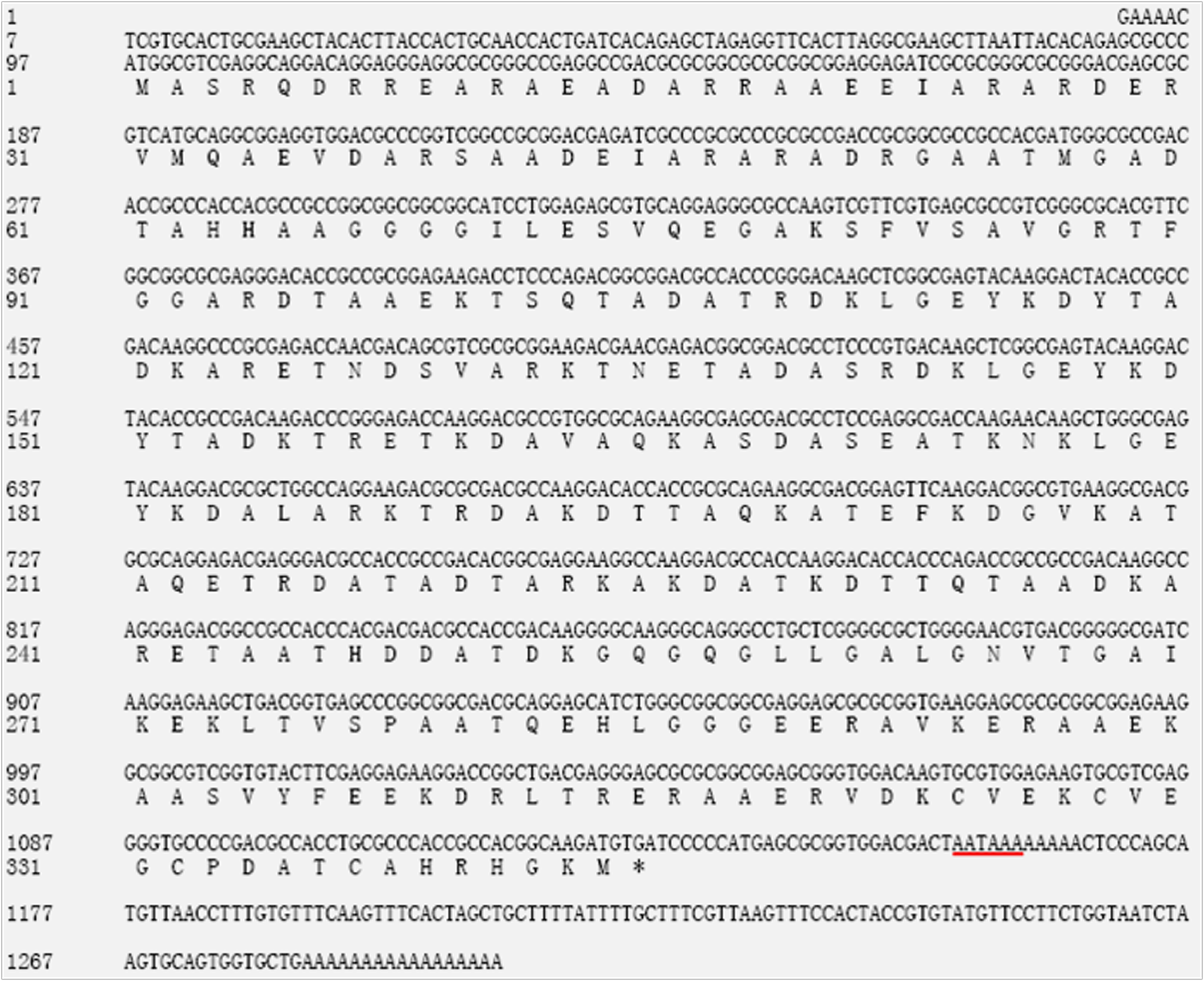

Supplement: Figure S1 — The OsLEA3-2 cDNA sequence and its deduced amino acid residues. The asterisk shows the stop code, the polyadenylation signal sequence (AATAAA) is underlined. The full length cDNA of OsLEA3-2 is 1,298 bp in size, with a 96 bp 5′ untranslated region (UTR) and a 167 bp 3′ untranslated region, and encodes an ORF of 344 aa. (TIF) [file pone.0045117.s001.tif]

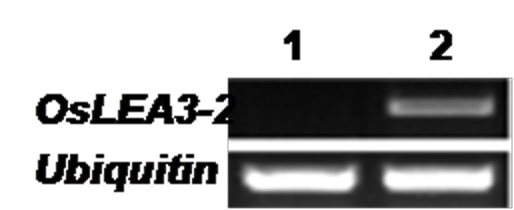

Supplement: Figure S3 — The expression profile of OsLEA3-2 in Oryza sativa. Lane 1, hongland; lane 2, hongland+10 µM ABA. (TIF) [file pone.0045117.s003.tif]

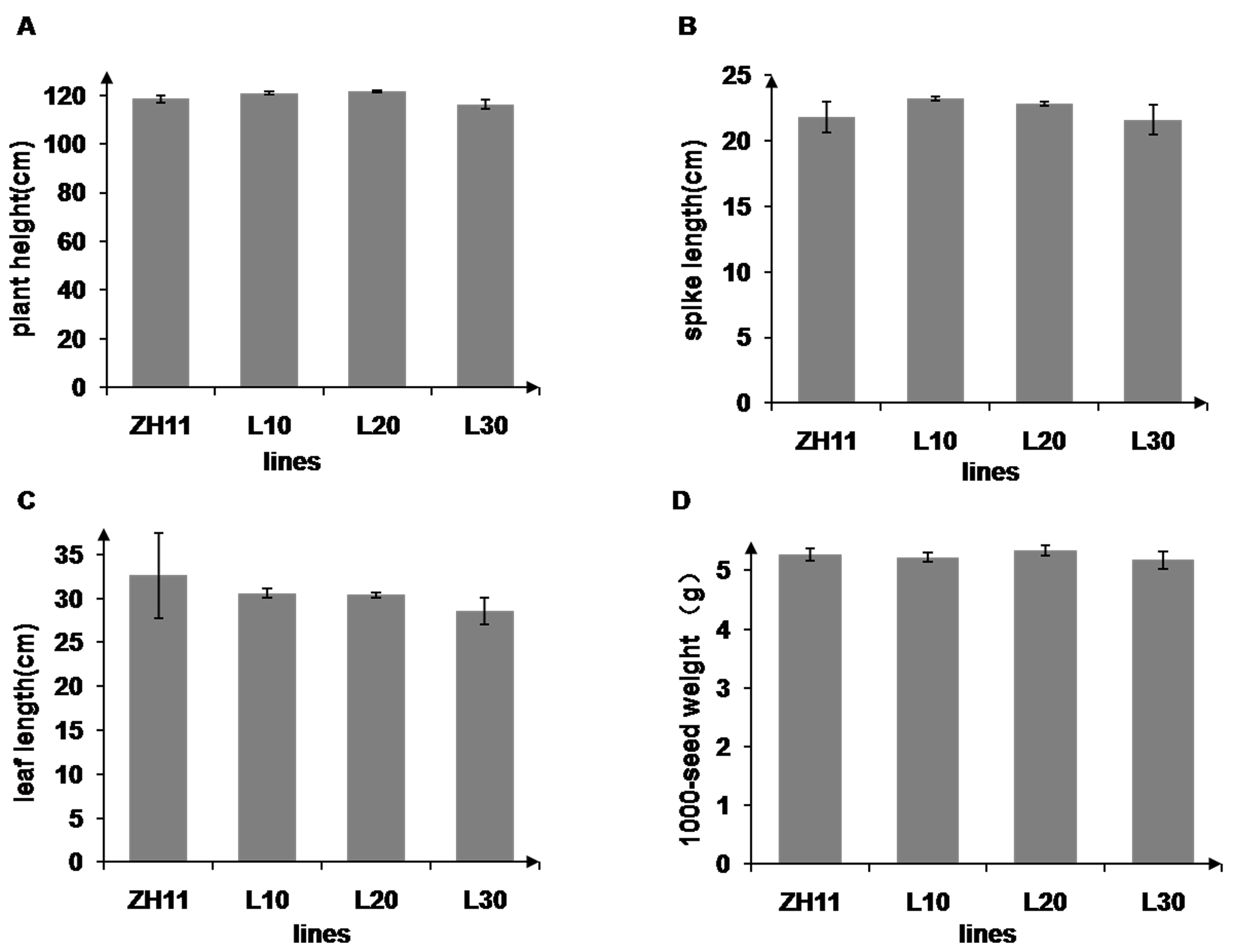

Supplement: Figure S4 — Plant height (A), spike length (B), leaf length (C), and 1,000-seed weight (D) of transgenic lines overexpressing OsLEA3-2 and wild type zhonghua 11 under field conditions with normal irrigation. Twelve samples were measured for plant height, spike length, and leaf length of each line. 1,000-seed weight was measured in triplicate. (TIF) [file pone.0045117.s004.tif]

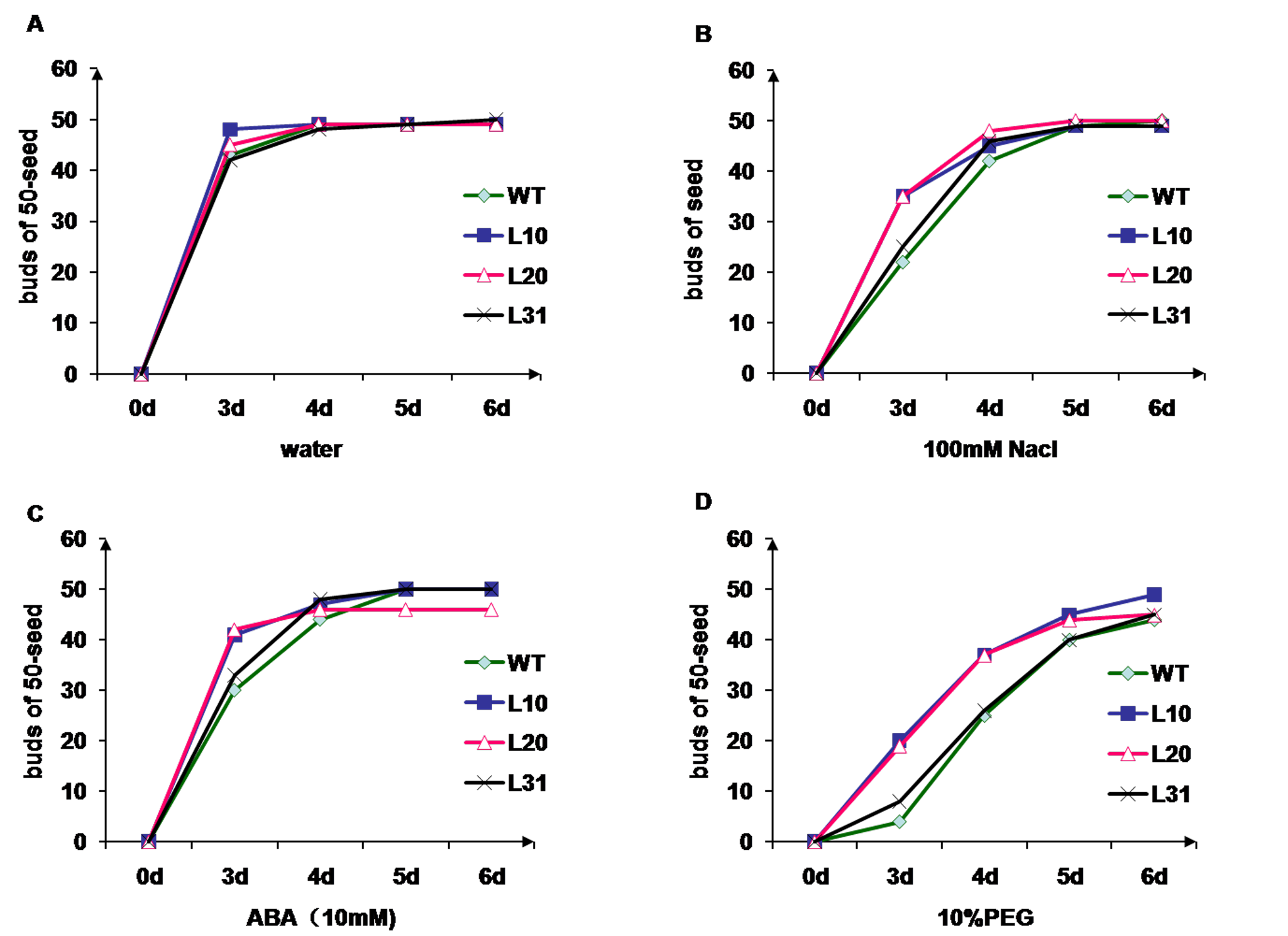

Supplement: Figure S5 — The germination of rice seeds in water (A), water+100 Mm NaCl (B), water+10 µM ABA (C), water+10% PEG (D) for 6 days. The germinated seed number was counted every day. The germination of rice seeds was assayed once. (TIF) [file pone.0045117.s005.tif]

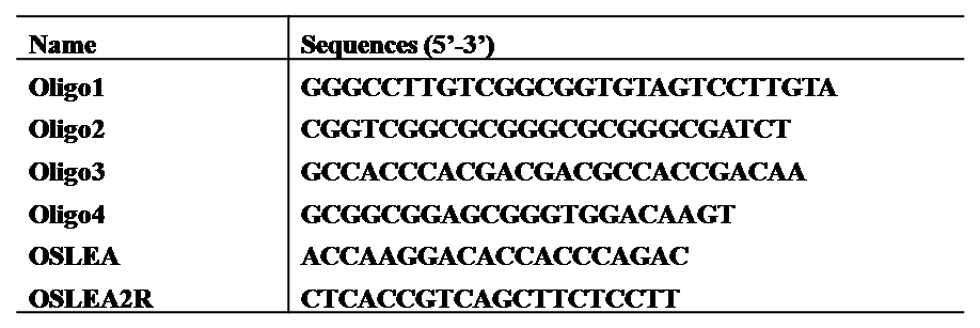

Supplement: Table S1 — Primers. (TIF) [file pone.0045117.s006.tif]
